# Psychological Problem Diagnosis and Management in the Geriatric Age Group

**DOI:** 10.7759/cureus.38203

**Published:** 2023-04-27

**Authors:** Ujwall Thakur, Anuj R Varma

**Affiliations:** 1 Psychiatry, Jawaharlal Nehru Medical College, Datta Meghe Institute of Medical Sciences, Wardha, IND; 2 Medicine, Jawaharlal Nehru Medical College, Datta Meghe Institute of Medical Sciences, Wardha, IND

**Keywords:** geriatric care, geriatric psychiatry, emergency department, geriatric medicine, geriatric depression

## Abstract

The definition of geriatrics is very complex to explain though it can be written as the treatment and care provided by healthcare and medical systems primarily to more venerable and senior citizens group of the population. The age group considered to be entering the old group is believed to be those who have reached their sixth decade of life. However, most of the global geriatric population doesn't need treatment until their seventh decade. Bodily impairment, both physical and mental, due to various reasons, for example, financial or personal reasons or feeling ignored, is reasonable for clinicians to anticipate caring for a growing proportion of older patients with complicated medical and psychosocial concerns. Complex ethical quandaries could develop as a result of these difficulties and problems. Who should anticipate ethical challenges faced by doctors early during management? We offer practical recommendations for improving communication because ineffective patient-clinician communication might result in moral dilemmas. Physical impairment, hopelessness, and cognitive decline are all more prevalent as people age. Politicians and healthcare providers of nations should step in to search for a measure to reduce the uprising of the condition; otherwise, it will lead to an uprising of the cases in an exponential manner. It is necessary to increase the financial challenges faced by the elderly. In addition, awareness should be increased, as well as programs aimed at enhancing their standard of living.

## Introduction and background

As the world ages, there is an increasing interest in varied morbidity patterns among the elderly. This study's central idea is to bring to light the problems and treatment of the conditions the elderly population goes through in their life [1]. Doctors are also very much concerned about the mental health of their patients for them to apply effective treatment to them. The success to be achieved by the treatment is, most of the time, very much dependent on the state of the patient. Therefore, who, regardless of the primary health condition for which the patient is receiving treatment, can never ignore it [2]? As a result, a doctor needs to know the proper method in solving the problem to provide aid in the treatment to the patient and improvement in primary care maintenance to comprehensive health facilities made accessible to the elderly. A geriatric mental study is a discipline of cognitive research in which the central point of attention is on the psychological issues that older people encounter. We are aging as an entire planet, not simply as ourselves. Aging will be a bigger problem for developing countries than for developed countries as they would have less time to adapt, and also, a shortage of resources would be more severe in developing countries than in developed countries. Aging is a natural part of the human experience. With the growth of medical research and technology in the twenty-first century, the mortality rate is decreasing, life expectancies are increasing, and fertility is decreasing [3]. There is an increase in the population aging trend. On the other hand, most people live to a ripe old age which may differ based on the development of their residing nation. It may be both a benefit and a burden in many ways. They may, for example, reach the age when they may pursue occupations, raise children, and enjoy the company of their grandkids. Unfortunately, old age brings with it its own set of issues. A person's body begins to wind down for its ultimate repose at this period of life. Many medical ailments, as well as familial and societal challenges, can have an impact on a person's thinking as they approach the end of their lives. In most cases, an individual's life is classified into four stages: young adulthood, adulthood, middle age, and elderly age. During these stages of life, individuals have to endure many difficulties to survive and maintain themselves. It is widely accepted that old age is a condition that no one wants, but it cannot be reversed or avoided.

Old age is also a difficult period of one's life. After the age of 65, aging problems are more common. These issues can be classified into five categories, namely physiological, psychological, emotional, financial, and social deterioration [4]. Mental and physical health are two of the most typical aging issues. Arthritis, heart disease, anxiety, and depression, for example, are more cases seen in the elderly compared to the average population. Medical and mental health specialists can treat or cure various senior issues, although degenerative diseases are more complex to treat, and palliative treatments are mostly given. Mental health professionals believe that depression among the elderly is a severe problem despite the widespread belief that the loss of interest, authority, and routine tasks are standard aging elements. Studies on senior healthcare emphasize the need for mental health treatment when older adults exhibit unhappiness, such as unexpected weight loss, a drop in grooming habits, and an inability to enjoy joy or pleasure. Memory loss and other impairments worry people, families, and societies worldwide. There is a great opportunity in treating these conditions through advancements in sciences; it has been shown that the onset of symptoms is much later than the beginning of the disease and that the incubation period is a great time for the treatment of the conditions. Despite all the promises made by the government media houses, preventing these conditions has not been adequate, and it is still a significant morbidity factor for the old age group [5]. Diagnosis and treatment of mental illnesses in older persons present a significant barrier to general care. Health programs suggest that high-income countries have a lower incidence of dementia than low-income countries [6]. Despite the lack of information on their usage for this purpose, electronic medical records have a lot of potential in this area. Infectious diseases are the major problem faced by the young population. Still, a shift can be seen toward chronic illnesses, which affect mental health during the later stages of life, as people live longer. Depression, anxiety, and substance misuse are the most prevalent mental illnesses of older individuals [7]. In addition to interfering with daily life and lowering the quality of life, mental diseases are linked to higher healthcare expenses, mortality, and suicide [8]. The policy emphasizes the significance of primary healthcare services to the mental health of older individuals. Though the knowledge of the primary health provider is enough for the essential treatment due to the frequency and experience in the study field of mental health of the elderly population, it is a barrier to treatment [9]. The situation today is becoming more and more severe for elderly patients worldwide. We need to address this situation, and to do it, we also need to adapt to new solutions that include new social and cultural programs for the old generation as the trend toward geriatric treatment facilities rises. In addition, medical, mental, and social facilities, as well as residential care facilities, should have a better treatment specialization. Aging brings both problems and opportunities.

## Review

Methodology

After reading several publications on psychological problems in the senior population, I wrote this review essay. The globally recognized medical databases were used as the search approach (mainly PubMed database). MeSH terms used in the article were "Aged, Geriatrics Assessments, Depressive Symptoms, and Emotional Depression." We looked at publications from a variety of journals that were published by renowned authors. We examined the referenced sources of the studies and corrected inaccuracies as needed. The data was sorted, and genuine literature was included in the review.

Psychiatric evaluation and identification in older adults

The occurrence of diseases and the progression of psychiatric disorders in the later stages of life is still very much unclear. This can be partially explained by the lack of research on the outcomes of psychiatric disorders in older persons [10]. These studies do, however, have some restrictions. When evaluating older persons, diagnostic challenges arise because general diseases accompany a person's behavioral and neurological symptoms. Still, old age can be due to other old-age conditions, and it's difficult to determine the exact etiology of conditions as old age brings many other co-morbid conditions. Additionally, cohort effects, changes in the body and the brain due to old age, and unusual symptoms of mental illnesses might result in erroneous or missed psychiatric diagnoses [11]. As with any medical or psychiatric assessment, getting a complete history is the best place to start. Any mental assessment must, of course, include a medical history. We shall, therefore, emphasize a few unique factors to consider while conducting an initial psychiatric assessment of older persons. Collateral information should always be considered, especially when patients show signs of cognitive deterioration [12].

History of presenting illnesses

When conducting psychiatric examinations of older persons, weightage should be given to this diagnosis in the differential diagnosis incidence of delirium and the incidence of psychiatric diseases due to medical conditions in late life [13]. Some situations could be apparent (e.g., confusion and hallucinations that come with a sign of pneumonia during the first two or three days of the occurrence of fever accompanied by cough and other symptoms), but many call for further caution (e.g., depression which is associated with high calcium levels due to a paranormal non-cancerous tumor of the parathyroid gland parathyroid adenoma) [14].

Types of disorders that come under geriatric psychological disorders and signs and symptoms

Signs and Symptoms of Senile Dementia

Individuals suffering from senile dementia show a lack/loss of memory, cannot tolerate changes in the environment, cannot maintain orientation, cannot sleep, lack judgment, have sudden episodes of delusions, are tired, have a high grade of depression, have a clouded mind that makes them restless, combative, resistant, and incoherent, eager to fight, and have illogical arguments. In difficult situations, they become bedridden, and their will to fight the illness decreases, reducing their life expectancy. The idea that dementia is a necessary component of aging is widely held among the general people (and even among professionals). Age is a major risk factor for dementia; it has been reported that at least 50% of people over the age of 85 still have normal cognitive function [15]. Undoubtedly, older persons are more likely to have poor decision-making skills. When addressing decreased decisional capacity, diseases like senile dementia, Alzheimer's, psychosis, anxiety, and phobia are considered. The emotional effects of psychosis and melancholy, such as paranoid delusions or extreme pessimism, can significantly affect an older person's decision-making ability. However, mental disease or dementia does not always compromise decisional ability [16,17]. Elderly people's cognitive abnormalities must be evaluated for symptoms such as depression, restlessness, insomnia, and irritability. Research is being done to develop accurate diagnostic classifications for these neuropsychiatric dementia symptoms [18].

Criteria

The criteria comprise a medical history that emphasizes cognition and function and comes from family, friends, or a caregiver. A brief bedside cognitive assessment should be done. Neuropsychological testing can also be advised if it is the necessary measure.

The Determinants of the Etiology of Dementia

Determining the etiology of dementia depends on the family history, neurological history, general and medical evaluations, physical examinations, neurological signs (e.g., cognitive impairment, focal examination, and other conditions), relevant symptoms for diseases of the vascular system or metabolic system, and neuropsychological evaluations.

Investigations

The standard workup typically consists of a few blood tests (such as B12 and TSH), and mainly imaging techniques, such as CT and MRI, are used to treat treatable causes [19]. After a brief history and physical examination, if the cause of dementia is still unknown, more extensive history and physical examinations, as well as certain blood, neurologic, and medical testing, should be considered. Cerebrospinal fluid (CSF) testing obtains evidence of positive cultures in infections, and in cases with hydrocephalus (water in the brain), after the removal of the excessive CSF fluid, the person whose gait was affected tends to walk normally [20].

Treatment

Reading and playing mentally taxing games like chess and bridge can help maintain cognition and function, as can participating in social activities like birthday celebrations, neighborhood chats, park walks, yoga, and light housekeeping chores [21,22]. They should always stay away from dangerous substances like firecrackers and poison. Driving a car or other vehicles should be avoided. Family members should always be supportive of them. A healthy diet that includes almonds, nuts, and berries is also beneficial [23]. See Table 1 for treatment options.

**Table 1:** Treatment options [24]

Name	Mechanism of action	Study population
Prazosin	Postsynaptic alpha-1 adrenoreceptor antagonist	Disruptive agitation in Alzheimer's disease/agitation/aggression in Alzheimer's disease
Cannabinoids	Ligands of cannabinoid receptors CB1 and CB2	Dementia-related agitation and aggression
Mirtazapine	Noradrenergic and specific serotonergic tetracyclic antidepressants (NaSSA)	Agitation in dementia
Lithium	Mood stabilizer: exact mechanism of action in mood regulation has not been clarified; inhibitor of glycogen synthase kinase 3B (GSK3)	Behavioral symptoms in frontotemporal disorders/psychosis and agitation in Alzheimer's disease
Escitalopram	Selective serotonin reuptake inhibitor	Agitation in Alzheimer's disease

Cerebral arteriosclerosis-related psychosis

Physiological symptoms include severe dyspepsia, unsteadiness in walking, and tiny strokes that result in cumulative brain damage and eventual behavioral changes. Seizures that are conclusive are rather common.

Signs and Symptoms

Signs and symptoms include weakness, tiredness, dizziness, headache, sadness, memory loss, bouts of disorientation, decreased job efficiency, increased irritability, and a propensity to be suspicious about little topics. In old age, general intellect and autonomous creative thinking are frequently harmed.

Investigation

An angiogram can be conducted, and remodeling the plaque further down the X-ray would look normal even though there is a very high percentage of narrowing present at the real site. MRI can calculate the quantity of the plaque and also helps in evaluating the composition. CT and other methods which can be useful in the diagnosis should also be considered.

Treatment

People with asymptomatic intracranial stenosis are frequently advised to take aspirin or other over-the-counter platelet inhibitors. Still, people who come with symptoms are given anti-coagulation drugs. The goal is to prevent further plaque development in asymptomatic individuals. They don't have any symptoms, but they probably will if there is more buildup. It is important to try to lessen the degree of stenosis in those exhibiting symptoms. The anti-coagulation drugs attempt to break down the existing buildup on the surface without an embolism while lowering the likelihood of subsequent buildup. Endovascular therapy is utilized for people with severe stenosis at risk for impending stroke [24].

Geriatric depression

Depression and anxiety are common geriatric disorders that have a negative effect on a person's balance (clumsy, unsteady), movement (involuntary movements like tremors can occur), and speech (racing thoughts and faster speech). Other aging issues, such as loss of taste, hearing, or smell, can be problematic, especially for seniors living alone. Deterioration in these vital senses makes older people increase the chances of accidents leading to more injuries. Depression is one of the risk factors for CVS disease, and it has been related to higher mortality rates. In addition, successful care of the main problem, who should undertake efforts to improve patients' psychological and social function, and more efforts should be put into improving issues related to the mental and emotional state of the person and relation to society's function.

The Geriatric Scale of Depression

The geriatric scale of measuring depression is a report measure of depression in older adults. It was the first scale used by the medium of 30 units of instrument. Since it was an ancient method and needed an update because it used to consume more and more time and there had been a delay in the measurement, things had to be practiced quickly. In addition, patients used to find this method difficult to complete; thus, a 15-unit update was created. A new update was the small geriatric scale of measuring, which is comprised of 15 units chosen from the older method that was named a large geriatric scale of measurement. The number 15 on the chart is of distinct values to one another. When the answer was given in a positive sense (10 on the scale), depression was declared, and when it was given in a negative sense (5 on the scale), depression was treated.

Signs and Symptoms

There are several warning signs and symptoms of depression, such as persistent sadness, anxiety, or sorrow, undiagnosed aches and pains that don't go away with medication, sudden appetite loss or weight loss, low motivation or energy, trouble falling asleep or staying awake, suicidal thoughts or prior suicide attempts, memory problems, forgetfulness, or difficulty making decisions.

Treatment

Drugs that are classified as second-generation antidepressants are used to treat older individuals particularly due to the decreased side effects of these drugs [25]. For these patients, having a thorough therapeutic index makes a prior choice easier: sertraline (Zoloft) 100-200 mg daily, venlafaxine (Effexor) 150-300 mg daily, paroxetine (Paxil) 20-40 mg daily, bupropion (Wellbutrin) 150-450 mg daily, duloxetine (Cymbalta) 20-120 mg daily, and escitalopram (Lexapro) 10-20 mg daily.

Parkinson's disease

Parkinson's disease is an extrapyramidal motor disorder that is characterized by the inability to bend, being forced out of shape, shaking rhythmic movements in one or more body parts, and being unable to be uncontrolled by one, as well as slowed or reduced muscle movements. Secondary features include defects in gait, posture, and dementia.

Signs and Symptoms

The most common age group to be affected is the old age group, which is around 60 years old. Signs and symptoms include shakiness, slowness of movement, hypertonia, disbalance, change in communication skills, change in handwriting, and age. Although less common than seniors, young adults can also be taken into account. It is a disease that worsens with advancing age and progresses.

Diagnosis

The diagnosis of Parkinson's disease is based on history and physical examination. Prodromal symptoms (such as rapid eye movement, sleep, behavioral disorders, hyposmia, and constipation), recognizable movement difficulties (such as tremors, stiffness, and slowness), and psychological or cognitive issues can all be present in the past (e.g., cognitive decline, depression, anxiety). Usually, an examination reveals bradykinesia with tremors, rigidity, or both. When the presence of parkinsonism is unclear, dopamine transporter single-photon emission CT can help in the diagnosis. There are various forms of Parkinson's disease, each with a unique prognosis. The disease progresses more quickly in those with a diffuse malignant subtype of Parkinson's disease (9% to 16% of patients), who also have strong early motor and nonmotor symptoms [26].

Treatment

A dopamine precursor (Levodopa) can be administered. Then, it is converted into the chemical dopamine, and dopamine is used up by the nerve fibers of the brain to communicate with each other. The following can also be administered: dopamine agonists (pramipexole, ropinirole, bromocriptine, cabergoline, and lisuride), COMT inhibitors (entacapone and tolcapone), MAO-B inhibitors (selegiline and rasagiline), and anticholinergic agents (benztropine and biperiden).

Alzheimer's disease

Alzheimer's disease is a progressive neurologic disorder that causes the brain to shrink (atrophy) and brain cells to die. This is a condition that worsens with time.

Signs and Symptoms

The early signs and symptoms of Alzheimer's disease are frequently misinterpreted and improperly diagnosed because they are mistakenly attributed to the patient's advanced age and stress-related conditions. The clinical criteria for the disease take up to eight long years; detailed neuro-physical testing might indicate moderate impairments. The beginning or early signals can also negatively affect a person's daily activities if they tend to forget their address and have trouble remembering their name. These are the most frequent signs of short-term memory loss, which show up as difficulties remembering information previously learned, an inability to remember new information, as well as a diminished capacity to learn new information [27]. Alzheimer's disease, the most common dementia cause, accounts for up to 80% of all dementia diagnoses [28].

Diagnosis

The National Institute on Aging and Alzheimer's Association updated the clinical criteria for the diagnosis of mild cognitive impairment (MCI) and the various phases of dementia caused by Alzheimer's disease. In 2011, by building on the original 1984 diagnostic criteria [29-30], the correct diagnosis of Alzheimer's disease requires that you be able to describe your symptoms and seek feedback from a close friend or family member on how they affect day-to-day life. Alzheimer's disease is also determined by the memory and cognitive tests doctors administer. The development of serum tests that can measure the number of circulating proteins linked to Alzheimer's disease is currently promising. In a small number of patients, one test in 2017 successfully distinguished between normal cognition, MCI, and dementia brought on by Alzheimer's disease with sensitivities and specificities of 84% and 88%, respectively [31]. The serum microRNA profile screen, which exhibited validity and reproducibility in smaller trials, is another blood test that exhibits potential [32]. Recently, a test that improves the diagnostic accuracy for Alzheimer's disease was developed using non-invasive diagnostic imaging [33].

Imaging and Laboratory

Testing can rule out additional potential causes and aid in the diagnosis of the illness producing the symptoms of dementia. Alzheimer's disease, mostly some years ago, was identified indelibly after death, after which the distinct plaques were seen. Nowadays, the availability of biomarkers and CSF test and scans, for example, PET scans, have also helped in diagnosing the conditions before.

Treatment

For people with Alzheimer's disease, there has been an addition of many novel pharmacological options, and two kinds of pharmacologic treatments available have been mentioned in this article. Patients with mild, moderate, or severe Alzheimer's disease and Parkinson's dementia are advised to take the cholinesterase inhibitors donepezil, rivastigmine, and galantamine as part of their treatment [34]. The nutritional supplement huperzine A has demonstrated advantages for cognitive function and daily living activities for patients who select alternative therapies [35]. Additionally, a lack of vitamin D has been linked to an increased risk of dementia from any cause, and patients with deficiencies are advised to take supplements [36]. In both cerebrovascular illnesses and neurodegenerative diseases, controlling cardiovascular risk factors contributes to overall brain health [37]. Inhibitors of cholinesterase can also be administered. These medications increase levels of cell-to-cell communication by conserving a chemical messenger that Alzheimer's disease depletes in the brain. These are frequently the first drugs used, and most patients report only slight symptom relief. Inhibitors of cholinesterase may help lessen neuropsychiatric symptoms, including agitation or depression. The cholinesterase inhibitors donepezil (Aricept), galantamine (Razadyne ER), and rivastigmine are frequently administered (Exelon). These medications' most common side effects include diarrhea, nausea, appetite loss, and sleep difficulties. Another medication that can be administered is memantine (Namenda). The advancement of symptoms in mild to moderate Alzheimer's disease is slowed by this medication, which acts on a different brain cell communication network, and can also be occasionally used along with a cholinesterase inhibitor. Disorientation and dizziness are two of the relatively infrequent side effects.

Etiology and risk factors

Geriatric age is the major etiological factor that we can consider, followed by histories of previous depression (having a family history of the associated disease), lack of social support (lack of family support for older adults as they are mostly neglected and have to spend most of their time alone and sometimes feeling like a burden to the family), any other co-morbidity associated with other various psychological problems, feeling ashamed of sometimes not being able to perform daily activities like going to the washroom or not being able to remember names, a financial crisis at an age where a person does not have an individual income and mainly depends on the money from their children, which can be depressing for someone independent all their life, overthinking (financial instability in the family can mentally affect the old generation, and they may hide their conditions in order to avoid the family having to pay for their medical expenses), and not able to spend more time with their grandchildren. The majority of old-age people who live alone in nursing homes experience depression due to a lack of family contact due to their families moving to a new location, such as from a rural to an urban area, completely altering their surroundings.

Specific Conditions That Should Be Regularly Checked

Geriatric people with genitourinary malignancies experience psychological, physical, and psychological sensitivity associated with age as a risk factor for problem gambling in older persons. Elderly dental care, geriatric patients' psychological and emotional needs during dental care, and senior citizens' alcohol use should all be examined. The elderly have social emergencies. When it coexists with a chronic medical condition, geriatric loneliness is a severe health risk that harms their health because depression is frequently accompanied by hypertension, coronary heart disease, and diabetes, and it may impact therapy and prognosis, making treatment more challenging and ineffective. Depression is not only a severe problem in and of itself but also exacerbates its other co-morbidities [38]. For elderly cancer patients, a comprehensive geriatric examination is required. When the brain ages with time, issues connected with health, brain pathology, and society and financial variables, such as the dissolution of support by family structures and loss of self-income, elderly persons are particularly vulnerable to mental morbidity. Dementia and mood disorders are two mental illnesses that are regularly seen. Other issues include physical and mental illnesses, addictions like abusing alcohol and tobacco, delusions, and mental psychosis [39].

The effects of COVID-19 on the geriatric population

Given that older persons have a higher risk of suicide than the general population, the circumstances surrounding COVID-19 might heighten this risk. The COVID-19 epidemic's social distancing policies and COVID-19 therapy's ethical guidelines may make the three key components of the Interpersonal Theory of Suicide, being excluded from society, feeling disposable, and being exposed to suffering, worse. The COVID-19 epidemic strains resources, and it has sparked moral discussions regarding practices involving prioritizing the treatment of younger populations. Such encounters can limit seniors' ability to obtain necessary medical and mental health treatments and may send harmful messages about frailty. Moreover, the COVID-19 pandemic's potential for long-term distress may have an impact on neurological, immune, and physiological functions, increasing the likelihood of suicide. There is a discussion of potential locations to increase alternative therapies and decrease social exclusion [40]. People with PTSD are more likely than the general population to have suicidal thoughts, attempt suicide, and possibly even die by suicide; nevertheless, individuals are far less likely to seek assistance due to stigmatization fears, the idea that symptoms will subside gradually, and occasionally an absence of understanding about the illness on its own [41]. In terms of mortality, COVID-19 infection has the greatest impact on the elderly, with 14.8% of cases occurring in adults over 80 compared to 0.2% in people under 40 [42]. More municipal governments are outlawing visits to care homes and long-term care facilities to reduce the risk of infection among elderly residents. Additionally, prohibiting communal TV viewing, board game play, and art therapy sessions may be detrimental to residents' mental and physical health. Residents of nursing homes are already prone to loneliness, and the absence of social connection brought on by a decline in visits from family and friends exacerbates the traumatizing effects of COVID-19 on their daily lives [43]. A difficult conundrum for patients, caregivers, and health professionals is sexual disinhibition in persons with neurocognitive impairments. The most typical change in sexual activity associated with the development of a neurobehavioral disorder is a downturn in sexual attraction; however, between 1.8% and 25.9% of patient samples with neurodevelopmental disorders have shown the onset of sexual dissociation or inappropriate sexual behaviors [44-46].

Discussion

Experts in gerontology, geriatric medicine, psychiatry, nursing, and social work, through the notion of a continuum of care, will provide medical, mental, social, and residential care and advocate changes in the medical and social service delivery systems. If recognized by observing the typical signs, geriatric depression can be easily avoided from becoming problematic. Who is responsible for treating the issue holistically with both pharmaceutical and non-pharmaceutical approaches? Avoid open-ended questions, especially if the older person has dementia or Alzheimer's disease. Give them undivided attention and patience if they can't communicate at a faster rate. Interrupt them as little as possible since they like to talk openly and from their hearts. In addition, avoid moving from one topic to another throughout the chat since this may cause them to become confused. By putting these suggestions into practice, the emergency department would also improve the care given to older people and be able to offer a better healthcare facility for them as a whole federation. Mental illnesses are frequently related to advanced age. Psychotic depressions are more common in the elderly. Senile dementia (associated with cerebral atrophy and degeneration) and psychosis with cerebral arteriosclerosis are the two most common psychotic illnesses in elderly adults (associated with either blocking or ruptures in the cerebral arteries). It has been estimated that these two illnesses account for almost 80% of psychotic disorders among the elderly in industrialized nations.

## Conclusions

Psychological problems in the senior age group, although not a severe disease, if no proper precautions are taken, may lead to severe complications. The symptoms are usually hard to detect. The common symptoms include trouble sleeping, loss of appetite, sudden weight loss, less motivation to work on daily activities, hard to remain awake, emotional meltdown, loneliness, and not wanting to communicate with other people around them. As I was going through the research, there was a significantly lower concentration on the treatment of the older part of the world's population when it comes to mental health. Primary-care physicians are, most of the time, the only people who have treated or can treat the older population group and, because of this reason, can cut short many problems that lay ahead in the life of those groups of population later on. The information in this article gives modern clinicians crucial resources they can use to deliver phychiatric care.
